# The expanding role of protease therapeutics (2012–2026): from replacement therapies to immune system modulation and beyond

**DOI:** 10.1042/BCJ20260152

**Published:** 2026-06-04

**Authors:** Sage E. Nelson, Samantha G. Martinusen, Raymond Pho, Lawton F. Long, Carl A. Denard

**Affiliations:** 1 Department of Chemical Engineering, University of Florida, Gainesville 32611, U.S.A.; 2 UF Health Cancer Center, University of Florida, Gainesville 32611, U.S.A.

**Keywords:** biotechnology, synthetic biology, therapeutics

## Abstract

Proteases are powerful therapeutic agents, offering unique advantages over conventional small molecules and biologics through their ability to directly and precisely cleave and (de)activate their protein targets. Since the early 1990s, FDA-approved protease therapeutics have served as replacement therapies for hematological disorders, as enzyme supplements for digestive disorders, and as treatments for neuromuscular disorders. Recent developments have expanded the use of native proteases to modulate antibody responses, improve transplant outcomes, and treat rare conditions with high unmet needs. Despite challenges such as immunogenicity and substrate specificity, the therapeutic landscape of proteases is being redefined by innovations in enzyme engineering and discovery. These advances, combined with targeted delivery strategies and improved stability, are reshaping proteases into precise and adaptable therapeutic agents. Rather than being limited to traditional uses, proteases are increasingly recognized for their potential to address complex conditions such as viral infections, neurodegeneration, and fibrosis, among others. With continued development, proteases are positioned to become a versatile and robust class of biologics with expanding clinical relevance. The present review explores the evolving landscape of protease therapeutics, focusing on their clinical applications, immune-modulatory capabilities, and future potential in precision medicine. This review provides a timely update to the comprehensive article “Proteases as Therapeutics” by Craik, Page, and Madison, published in the Biochemical Journal in 2011.

## Introduction

The ability to harness proteases as precise therapeutic agents has long been a compelling goal in medicine. Early perspectives, including a prominent 2011 review by Craik and colleagues, described proteases as a distinct therapeutic class with diverse clinical applications [1,2]. Since then, a growing number of proteolytic enzymes have been approved for clinical use, primarily as enzyme replacement therapies or as agents that act on well-defined extracellular substrates (Table 1). These include treatments for hematological and metabolic disorders as well as drugs that degrade extracellular protein targets, such as ADZYNMA® for congenital thrombotic thrombocytopenic purpura and Voraxaze® for reducing toxic plasma methotrexate levels. Thus far, FDA-approved protease-based therapies have predominantly exploited only a subset of proteases' native biological capabilities.

**Table 1:** FDA-approved protease drugs from 2011 to 2025

Drug name	Developer	Protease	Protease type	Usage	Year of FDA approval
Xigris® (Drotrecogin alfa)	Eli Lilly and Company	Human activated protein C	Serine	Treatment of severe sepsis	2001 (withdrawn in 2011)
Ultresa®	Allergan	Pancrelipase	Serine	Exocrine pancreatic in-sufficiency	2012 (discontinued in 2016)
Viokace®	Aptalis Pharma (currently distributed by Aimmune Therapeutics)	Pancrelipase	Serine	Exocrine pancreatic insufficiency	2012
Pertzye®	Digestive Care, Inc.	Pancrelipase	Serine	Exocrine pancreatic insufficiency	2012
Jetrea®	Oxurion	Ocriplasmin	Serine	Vitreomacular adhesion	2012 (discontinued in 2020)
Alprolix®	Sanofi	Anti-hemophilic factor IX-Fc fusion protein	Serine	Hemophilia B	2014
Idelvion®	CSL Behring	Anti-hemophilic factor IX albumin fusion protein	Serine	Hemophilia B	2016
Coagadex®	Bio Products Laboratory (acquired by Kedrion Biopharma)	Coagulation Factor X	Serine	Hereditary factor X deficiency	2015
Brineura® (Cerliponase alfa)	BioMarin Pharmaceutical	Tripeptidyl-peptidase 1 (TPP1)	Serine	Neuronal ceroid lipofuscinosis type 2 (CLN2)	2017
Xiaflex®	Endo, Inc.	Combination of bacterial collagenases	Metalloprotease	Dupuyter's contracture	2010
				Peyronie's disease	2013
Adzynma®	Takeda Pharmaceuticals	ADAMTS13	Metalloprotease	Congenital thrombotic thrombocytopenic purpura	2023
Nexobrid®	Vericel	Mixture of proteolytic enzymes from pineapples	Cysteine	Eschar removal	2022
Voraxaze®	Serb Pharmaceuticals	Glucarpidase	Carboxypeptidase	Treatment of toxic plasma methotrexate concentrations	2012

A central objective in next-generation protease therapeutics is therefore to move beyond enzyme replacement and instead draw inspiration from the regulatory roles proteases play *in vivo*. Proteases regulate a myriad of cellular processes, from blood coagulation and protein activation/degradation to host defense and adaptive immunity [3,4]. Importantly, they achieve this regulation not by completely degrading proteins, i.e., proteasome enzymes, but by cleaving specific sequences within their targets. Consequently, proteases offer a unique opportunity to selectively eliminate disease-driving proteins, rewire dysregulated biological pathways, or augment innate host defense mechanisms [5–7]. Moreover, compared with antibodies, which are highly effective for systemically accessible targets but act in a strictly stoichiometric manner, enzymes offer a fundamentally different pharmacologic paradigm. As enzymes, proteases can turn over multiple substrate molecules, enabling sub-stoichiometric activity that is particularly well-suited for high-abundance targets or sites that are poorly accessible from circulation [8]. Furthermore, emerging approaches such as mRNA-based delivery of proteases aim to enable transient *in vivo* production of enzymes like ADAMTS13, potentially reducing the need for repeated protein infusions, although these strategies remain in preclinical development [9].

**Figure 1:**
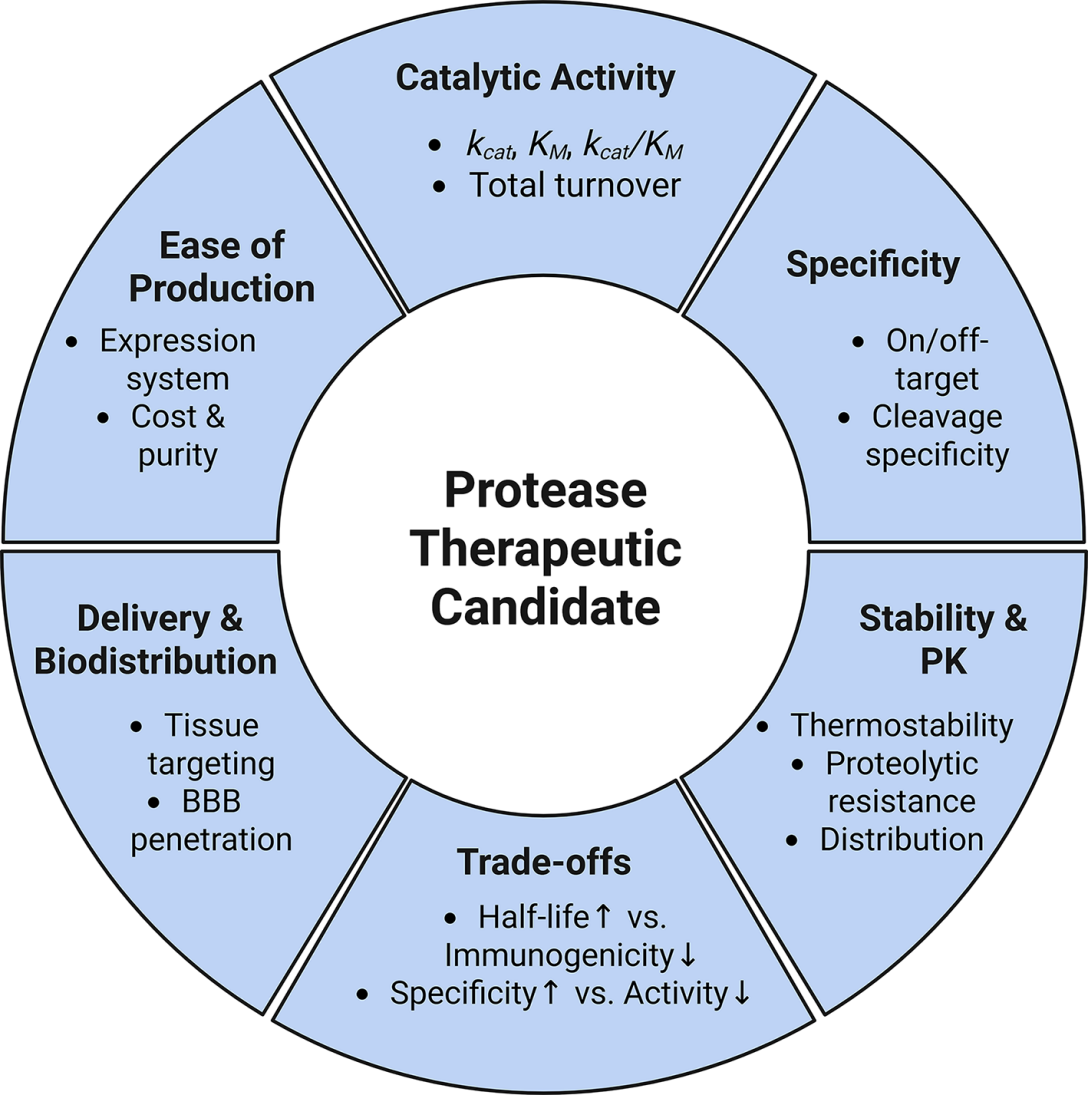
Overview of the factors governing the clinical translation of protease therapeutics including: catalytic activity, specificity, stability/pharmacokinetics, trade-offs, delivery/biodistribution, and ease of production

Despite this considerable therapeutic potential, successful clinical translation of any protein-based therapeutic requires overcoming a broad and interconnected set of engineering and pharmacologic challenges. For proteases, these considerations include catalytic performance (*k*_cat_/*K*_M_), substrate specificity, immunogenicity, stability (resistance to proteolysis), delivery constraints, and manufacturability (Figure 1) [2,8,10]. In parallel, pharmacokinetic properties, such as clearance rate and bioavailability, are critical determinants of dosing feasibility. While many protease therapeutics are administered intravenously to ensure systemic exposure, alternative routes including oral delivery (e.g., proteases targeting dietary antigens such as gluten), topical application, and localized injection have also demonstrated clinical potential [2,11]. Manufacturing considerations likewise play a critical role, as recombinant proteases produced in bacterial or eukaryotic systems must meet stringent purity standards, including rigorous removal of host cell proteins and other contaminants [12]. These requirements contribute significantly to production costs and can influence platform selection during early development. Beyond manufacturing, optimization of protease therapeutics often requires balancing improvements in pharmacokinetic and biophysical properties against preservation of catalytic function. For example, strategies to improve serum half-life, such as PEGylation or Fc fusion, can reduce catalytic efficiency or substrate accessibility, highlighting a fundamental modification–activity trade-off [13–15]. Similarly, achieving high specificity often comes at the expense of catalytic rate or stability, necessitating multi-parameter optimization during development [16,17]. Collectively, these constraints underscore the need for integrated engineering approaches that simultaneously address activity, specificity, stability, and delivery [16,18].

Within this multidimensional framework, substrate specificity emerges as a particularly important determinant of clinical tractability. A key throughline in the early expansion of the protease pharmacopeia is its reliance on the limited subset of proteases that possess inherently narrow substrate specificities [19,20]. To fully harness the therapeutic potential of proteases, there is a pressing need to overcome the longstanding challenges associated with engineering protease specificity. Recent advances in experimental and computational protein engineering, including AI-driven approaches for *de novo* protease design, are rapidly expanding the space of proteases with tailored activity and specificity. These advances are widely viewed as a major driver of innovation in the field [21]. Diffusion-based generative models and related frameworks have begun to overcome long-standing limitations in scaffold selection and specificity engineering, accelerating protease discovery and optimization [22]. These methodological advances, together with recent progress in protease specificity engineering, have been comprehensively reviewed elsewhere [18]. Accordingly, rather than focusing on protease engineering platforms, the present review centers on how proteases are being deployed in recent clinical, preclinical, and emerging therapeutic applications.

As the present review highlights, recent progress in protease therapeutics has been driven less by a single technological advance than by convergent innovation across multiple application domains. In particular, three emerging areas are reshaping the landscape of protease-based medicines: (1) bacterial proteases that cleave immunoglobulins to modulate immune responses and treat autoimmune disease; (2) microbial and plant proteases that selectively degrade pathogenic proteins, including dietary antigens such as gluten and disease-associated substrates implicated in neurodegeneration; and (3) engineered proteases designed to destabilize misfolded or aggregated proteins. Together, these examples illustrate how advances in targeting, specificity, and delivery are enabling proteases to move beyond replacement therapies toward more precise and programmable therapeutic modalities.

## Advancing hematological therapies through engineered proteases

Protease-based therapeutics have a long history in the treatment of hematological disorders. Early clinical successes include the replacement of essential coagulation proteases such as factor IX (FIX). Recombinant FIX, marketed as BeneFIX®, was approved by the FDA in 1997 and is effective at treating acute bleeding episodes [2]. Despite its success, FIX replacement therapy requires repeated dosing due to a relatively short circulating half-life (∼18 h), which increases the risk of developing anti-FIX antibodies.

One strategy to address these limitations is to extend protease residence time in circulation by engineering stabilizing molecular interactions. In the coagulation system, fusion of factor VIII (FVIII) to a high-affinity anti–von Willebrand factor (VWF) nanobody (Nb) enhanced FVIII–VWF complex stability by slowing dissociation kinetics, resulting in prolonged circulatory residence time while preserving normal thrombin-mediated proteolytic activation [23]. *In vivo*, this increased complex stability translated into improved hemostatic efficacy and a substantial reduction in anti-FVIII antibody formation. Conceptually, this approach parallels other circulation-time extension strategies, such as albumin binding, but uniquely leverages a native physiological interaction to improve both pharmacokinetics and immunogenicity.

In the past decade, the hematology therapeutic landscape has broadened substantially with the introduction of antibody-based agents and factor-mimetic biologics, including bispecific antibodies that can functionally replace missing coagulation factors [24–26]. These modalities have delivered transformative clinical benefits and now constitute a major focus of therapeutic development. Importantly, however, their emergence has not marked a departure from protease-based strategies. Rather, ongoing efforts to engineer coagulation proteases, such as improved variants of activated recombinant factor VII, reflect continued innovation within the field.

Marzeptacog alfa (activated) (MarzAA) is a variant of activated recombinant human FVII developed for the treatment of episodic bleeding in patients with inherited bleeding disorders [27]. MarzAA has four site-directed amino acid substitutions. Two catalytic-domain mutations (Q286R and M298Q) enhance platelet binding and tissue factor–independent activation of factor X, increasing procoagulant activity [28]. Two additional substitutions (T128N and P129A) introduce an N-linked glycosylation site to extend the circulating half-life [29]. Across two phase I studies (NCT01439971 and NCT04072237) and one phase II trial (NCT03407651), MarzAA demonstrated a favorable safety and pharmacokinetic profile following both intravenous and subcutaneous administration, with no serious adverse events or antidrug antibody formation reported [27,29,30]. In a phase II prophylaxis study in patients with hemophilia A/B with inhibitors, daily subcutaneous MarzAA (30 μg/kg with escalation to 60 μg/kg for breakthrough bleeding) produced a marked and statistically significant reduction in annualized bleeding rate, decreasing from a baseline mean of approximately 19.8 to 1.6, along with a substantial reduction in the proportion of days with bleeding. Despite these results, the subsequent phase III study (NCT04489537) was terminated after Catalyst Biosciences discontinued development due to feasibility constraints, including enrollment challenges, increased competition, and widespread adoption of alternative prophylactic therapies [31]. MarzAA was later acquired by GC Biopharma, and its future approval depends on its success in clinical trials [32].

Beyond MarzAA, Catalyst Biosciences has leveraged its engineering platform to advance additional protease therapeutics, including dalcinonacog alfa for subcutaneous treatment of rare bleeding disorders, and CB 4332, an engineered complement factor I (CFI) for systemic prophylaxis in patients with CFI deficiency [33]. However, dalcinonacog alfa was transferred to GC Biopharma as part of the same acquisition as MarzAA in 2023, while catalyst’s complement portfolio, including CB 4332 and related intellectual property, was sold in 2022 to Vertex Pharmaceuticals [32,34].

Despite strong mechanistic rationale and compelling clinical efficacy, the MarzAA program illustrates how advances in protease engineering can be constrained by translational and market forces rather than biological feasibility.

## Modulating immune responses through proteolytic deactivation of immunomodulators

Autoimmune diseases encompass a broad spectrum of conditions characterized by dysregulated B-cell and T-cell function [35]. Many of these autoimmune diseases, including systemic lupus erythematosus (SLE) and rheumatoid arthritis (RA), are associated with elevated levels of immunoglobulin M (IgM) and immunoglobulin G (IgG) autoantibodies [36]. Current therapeutic strategies focus primarily on broad immunosuppression using agents like corticosteroids, non-steroidal anti-inflammatory drugs, and disease-modifying antirheumatic drugs, but these treatments often come with severe side effects and incomplete disease control [37]. Biologics have made some progress in addressing these challenges. For example, belimumab, a monoclonal antibody targeting B-cell activating factor, can reduce disease activity and organ damage in SLE [38]. However, due to the heterogeneity of IgG-mediated disease, belimumab has limited efficacy, and it is currently not approved for patients with severe active central nervous system lupus [39].

Against this backdrop, proteases are emerging as precision immunomodulators. By selectively degrading immunoglobulins, cytokines, and other immune mediators, proteases can dampen inflammation and limit immune-mediated damage in autoimmune diseases, organ transplantation, and celiac disease. To clarify how proteases can be harnessed in this way, it is useful to consider the shared and divergent features of the major immunoglobulin classes.

Immunoglobulins comprise multiple classes (IgM, IgG, IgA, IgE, and IgD) that share a conserved overall architecture but differ in quaternary structure, localization, and effector function [40]. All antibodies contain two antigen-binding Fab regions and a constant Fc region; however, differences in heavy-chain constant domains give rise to distinct isotypes and subclasses. IgG, the most abundant serum antibody, is further divided into subclasses (IgG1 to IgG4) that vary in hinge length, disulfide bonding, and neonatal Fc receptor (FcRn) interactions. IgA exists primarily as a dimer in mucosal tissues, while IgM forms pentameric structures that enable high-avidity binding. Despite these differences, conserved sequence and structural elements provide accessible cleavage sites that proteases can exploit. For example, IgG-cleaving enzymes such as IdeS recognize conserved motifs within the lower hinge region, enabling broad activity across subclasses, whereas other proteases exhibit selectivity based on subtle sequence or structural differences between isotypes [41]. These shared and divergent features underline how proteases can achieve either pan-immunoglobulin activity or isotype-specific targeting.

Several of these enzymes are now in clinical trials to evaluate their safety and efficacy (Table 2). The following subsections highlight key proteases targeting IgG, IgM, IgA, and immunogenic gluten peptides, outlining their mechanisms, therapeutic promise, and translational challenges.

**Table 2:** Protease drugs in development

Drug name	Developer	Protease	Protease type	Usage	Target protein or pathway	Development status
Imlifidase or (Idefirix®)	Hansa BiopharmaIgG	IgG-degrading enzyme	Cysteine	Desensitization in organ transplantation	IgG cleavage	Biologics license application (BLA) accepted as of 2026
HNSA-5487	Hansa Biopharma	IgG-degrading enzyme	Cysteine	Desensitization in organ transplantation	IgG cleavage	First in human (FIH) study completed in 2024
S-117	Seismic Therapeutics	IgG-degrading enzyme	Cysteine	Desensitization in organ transplantation	IgG cleavage	Phase I as of 2025
CYR212	Cyrus Biotechnology	IgG-degrading enzyme	Cysteine	Treatment of autoimmune diseases	IgG cleavage	Preclinical as of 2025
KJ103	Shanghai Bao Pharmaceuticals Co., Ltd.	IgG-degrading enzyme	Cysteine	Anti-glomerular basement membrane (anti-GBM) disease	IgG cleavage	Phase II as of 2025
S-8484	Seismic Therapeutics	IgE-degrading enzyme	Cysteine	Treatment of allergic diseases	IgE cleavage	Investigational New Drug (IND)-enabling studies as of 2025
Latiglutenase (ZMGX003)	ZymagenX	Mix of EP-B2 and PEP	Cysteine	Celiac disease	Enzymatic breakdown of gluten	Phase II as of 2022
HTI-501	Halozyme Therapeutics	Conditionally active recombinant human cathepsin-L	Cysteine	Dermatological applications	Lysosomal enzyme replacement therapies	Phase II as of 2013
DM199 (rinvecalinase alfa)	DiaMedica Therapeutics	Recombinant human kallikrein-1	Serine	Acute ischemic stroke	Improves blood flow to ischemic areas	Phase II/III as of 2025
			
				Preeclampsia and fetal growth restriction	Improves endothelial health by stimulating beneficial signaling pathways	Phase II as of 2025
ANG003	Anagram Therapeutics, Inc.	Mixture of lipase, protease, and amylase. The protease is a serine protease produced in *Aspergillus melleus*	Serine	Exocrine pancreatic insufficiency	Breaks down food into more easily absorbable components	Phase II as of 2026

### Harnessing IdeS for immune modulation and transplant success

**Figure 2:**
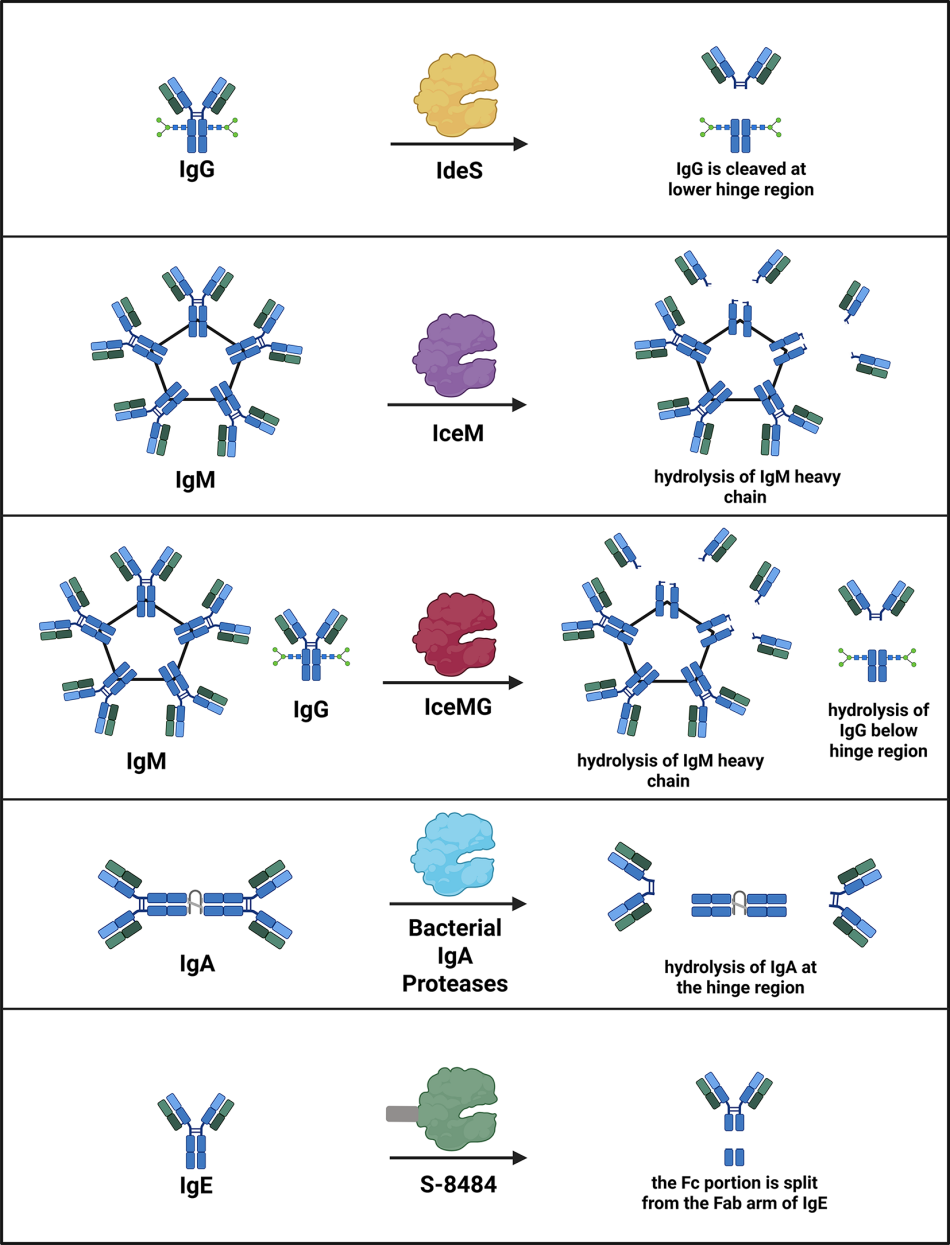
Proteolytic inactivation of immunoglobulins as a strategy to modulate immune responses. Diverse immunoglobulin-targeting proteases cleave IgG, IgM, and IgE at distinct structural sites, generating antibody fragments with characteristic functional outcomes. For example, IgG-degrading enzymes such as IdeS and its engineered derivatives cleave the IgG hinge region, removing Fcγ-mediated effector functions and disrupting FcRn and complement activation. Together, these proteases illustrate how selective cleavage of immunoglobulin isotypes can rapidly and precisely suppress pathogenic humoral immunity across autoimmunity, transplantation, allergy, and gene therapy.

IdeS (imlifidase), an IgG-degrading cysteine endopeptidase from *Streptococcus pyogenes*, cleaves the Fc region of IgG antibodies, blocking FcRn interactions and interfering with immune activation (Figure 2) [42,43]. Repurposed for desensitization in transplantation, IdeS reduces donor-specific antibodies (DSAs) in highly sensitized patients at risk of graft rejection due to pre-existing antibodies against human leukocyte antigens (HLAs) [44]. In one study, 24 of 25 such patients underwent successful HLA-incompatible kidney transplantation with stable renal function maintained post-surgery treatment [43].

However, since IdeS is derived from a bacterial pathogen, it can trigger the formation of anti-drug antibodies (ADA) and the non-selective cleavage of protective antibodies [45]. Most patients have low-level preexisting anti-IdeS IgG from prior *S. pyogenes* exposure and develop an IgG response that typically peaks 2 to 3 weeks after treatment [46]. IdeS also has a short half-life (∼5 h), which contributes to a significant limitation: donor-specific HLA antibodies and anti-HLA antibodies often rebound within 3 to 7 days, frequently requiring concurrent immunosuppressive therapy [15,44]. To address IdeS’ short half-life, a monovalent Fc fusion variant (IdeS-Fc^monov^) was engineered by genetically fusing IdeS to a heterodimeric human IgG Fc [15]. The Fc fusion extends serum persistence via FcRn-mediated recycling while minimizing Fc effector function [47]. The Fc domain was engineered to be monovalent and inert, preventing unwanted immune activation and protease-mediated self-cleavage. This construct increased circulating half-life approximately seven-fold; however, steric and conformational constraints introduced by the Fc fusion reduced catalytic efficiency relative to wild-type IdeS, highlighting a trade-off between pharmacokinetic enhancement and enzymatic activity [15].

Despite these limitations, IdeS received conditional approval from the European Medicines Agency (EMA) in 2020 to treat kidney transplant recipients with a positive crossmatch [48]. A five-year study of 39 patients showed 90% patient survival and 84% graft survival [49]. More recently, ConfIdeS, a Phase 3 trial conducted by Hansa Biopharma, evaluated 64 highly sensitized kidney transplant patients [50]. Patients randomized to receive IdeS exhibited markedly superior kidney function at 12 months, with a mean estimated glomerular filtration rate of 51.5 ml/min/1.73 m^2^ compared with 19.3 ml/min/1.73 m^2^ in the control arm.

Beyond transplantation, IdeS is in Phase 3 evaluation for anti-glomerular basement membrane (anti-GBM) disease (GOOD-IDES-02 trial) and holds orphan drug designation from both the U.S. FDA and EMA [51]. Hansa Biopharma’s IgG-cleaving enzyme, HNSA-5487, achieves >95% IgG reduction in hours, with a significantly lower ADA response compared with IdeS, suggesting improved potential for redosing [52]. HNSA-5487’s favorable safety profile suggests its potential application in chronic autoimmune diseases, including myelin oligodendrocyte glycoprotein antibody disease, neuromyelitis optica, and myasthenia gravis.

### Targeting IgM to overcome immune barriers in gene delivery

Recombinant adeno-associated virus (rAAV) gene therapies face immunological hurdles from IgM and IgG antibodies [53]. rAAV vectors are the leading platform for gene delivery therapy in a variety of diseases, including hemophilia A, hemophilia B, and Crigler–Najjar syndrome [54]. However, neutralizing antibodies against AAVs are present in 30% to 60% of humans, and even low titers can block AAV gene transfer [55]. IgG-cleaving proteases, such as IdeS, IdeZ, and IdeXork, have been explored to overcome anti-AAV immunity and transiently deplete circulating IgG [56,57]. While these enzymes can create a brief window of reduced IgG levels, their efficacy diminishes at higher neutralizing titers, and the rapid development of ADA drastically reduces the effectiveness of redosing attempts [57].

To overcome the IgM-mediated anti-AAV immune response, IceM, an IgM-specific protease from *Lachnoanaerobaculum saburreum*, was recently identified and optimized [58]. To broaden its utility, a dual-activity enzyme, IceMG, was engineered by fusing IceM to an IgG-cleaving domain. Preclinical studies demonstrate that IceM and IceMG transiently degrade circulating IgM and IgG, effectively mitigating complement activation, a key driver of AAV vector clearance [58]. Notably, IceMG treatment reduced AAV9-induced complement activation within an hour and sustained its effect for up to two weeks, suggesting a potential therapeutic window for preconditioning prior to gene therapy. These proteases may also be useful in the context of autoimmune disease and transplantation. For example, a subset of myasthenia gravis (MG) involves IgM autoantibodies that contribute to disease pathogenesis, meaning IdeS alone would be insufficient, and combination therapy with an IgM-cleaving protease or the engineered IceMG enzyme may be optimal [59]. However, because IgM plays an important role in immune homeostasis by supporting apoptotic cell clearance, limiting excessive inflammation, and regulating autoimmunity, careful safety evaluation will be necessary before advancing these therapeutics toward clinical use [60].

### Expanding immunoglobulin targeting with engineered proteases from Seismic Therapeutics

Seismic Therapeutics, a clinical-stage biotechnology company leveraging machine learning-guided design through its IMPACT platform, is advancing a pipeline of protease therapeutics to selectively cleave disease-relevant immunoglobulins. Two lead candidates, S-1117 and S-8484, exemplify this approach by targeting IgG and IgE, respectively [61]. S-1117 is a novel engineered Fc-fused pan-IgG protease designed to address IgG autoantibody-mediated diseases [61]. To improve pharmacokinetics while minimizing unintended immune activation, Seismic Therapeutics linked the protease to a human IgG1 Fc domain that is specifically modified to lack Fc-mediated effector function and resist proteolytic self-cleavage. This design extends serum half-life through FcRn-mediated recycling while maintaining catalytic activity against IgG substrates [59]. Seismic also used their proprietary IMPACT platform to decrease immunogenicity by reducing T and B cell epitopes. Preclinical studies show that S-117 cleaves all IgG subclasses, removes IgG effector functions, and can also cleave membrane-bound IgG on memory B cells [62]. With its reduced immunogenicity and improved half-life, S-1117 may support both acute and chronic dosing, unlike IdeS, which is generally limited to single or infrequent administration. In March 2025, Seismic initiated a randomized, double-blind, placebo-controlled Phase 1 trial (NCT06828393) to evaluate the safety, tolerability, pharmacokinetics, and pharmacodynamics of S-1117 in healthy volunteers.

In parallel, Seismic is advancing S-8484, an engineered Fc-fused IgE protease designed to treat allergic diseases [63]. IgE plays a central role in type I hypersensitivity disorders such as food allergy, asthma, and allergic rhinitis. Furthermore, IgE-mediated food allergy is a major driver of anaphylaxis in children [64]. S-8484 was optimized for selectivity and potency using structure-augmented machine learning, overcoming the promiscuity often associated with natural bacterial proteases. Preclinical *in vitro* studies demonstrated rapid and selective cleavage of soluble IgE, depletion of IgE from B-cell receptors, and down-regulation of high-affinity IgE receptors on mast cells from humanized mice [63]. *In vivo* S-8484 reduced serum IgE and eosinophils in allergen-induced asthma models, attenuated IgE-driven tissue inflammation, and provided rapid resolution of systemic anaphylaxis. These data support ongoing IND-enabling studies and highlight the potential for a protease-based therapeutic to induce durable IgE depletion across multiple allergic indications.

### Transforming IgA nephropathy treatment with IgA-degrading proteases

Aberrantly glycosylated immunoglobulin A (agIgA1), a key pathogenic factor in IgA nephropathy (IgAN), arises from aberrant O-glycosylation in the hinge region of IgA1 [65]. These agIgA1s can be recognized by circulating IgG or IgA antibodies, promoting the formation of immune complexes that then trigger inflammation and glomerular damage [66]. Bacterial IgA-cleaving proteases from strains such as *Neisseria gonorrhoeae*, *Haemophilus influenzae*, and *Neisseria meningitidis* specifically degrade agIgA1 without affecting other proteins like human IgG or bovine serum albumin [65]. Excitingly, *in vitro* and mouse model studies demonstrate effective degradation of agIgA1 and its immune complexes. Nonetheless, further studies are needed to ensure enzyme specificity, safety, and efficacy in advanced models before clinical translation.

Mechanistic studies of IgAN show that circulating agIgA1 forms complexes with soluble receptor CD89 and anti-agIgA1 autoantibodies. These complexes are retained in the mesangium via interactions with the transferrin receptor, triggering complement activation, inflammation, and progressive glomerular injury [67]. In a humanized mouse model of IgAN, recombinant IgA1-cleaving proteases rapidly reduced circulating IgA1–CD89 complexes and markedly decreased mesangial IgA1 deposition following systemic administration. Repeated dosing further disrupted associated mesangial binding partners, including CD89, transferrin receptor, and transglutaminase 2, leading to reduced complement deposition, inflammatory cell infiltration, fibrosis, and hematuria, thereby demonstrating disease-modifying potential.

Notably, in addition to cleaving IgA1, the meningococcal IgA1-specific serine protease can also degrade human IgG3 [68]. Despite its low abundance in serum (∼7%), IgG3 plays a critical role in antibacterial immune defense, and reduced IgG3 levels are associated with severe bacterial respiratory tract infections [68,69]. These findings underscore the need to carefully evaluate off-target effects when considering IgA1-P as a therapeutic strategy.

### Protease supplementation to combat celiac disease

Celiac disease (CeD) is driven by immunogenic gluten peptides resistant to degradation by human digestive enzymes [70]. Bacterial and fungal-derived gluten-degrading enzymes, particularly prolyl endopeptidases (PEPs), show promise in degrading these peptides but face challenges like instability in gastric conditions and incomplete efficacy *in vivo* [70,71]. Chemical modifications such as PEGylation can enhance stability but may reduce activity or increase immunogenicity [72].

In contrast, a novel endoprotease 40 (E40), derived from the acidophilic actinomycete *Actinoallomurus* A8, exhibits robust glutenase activity at gastric pH and can resist pepsin degradation, effectively detoxifying gluten peptides *in vitro* [73,74]. Proteomic and immunoassay analyses confirmed that E40-treated gastric digestion exhibited a marked reduction in α-gliadin epitopes, known to trigger immune responses in CeD patients. Importantly, residual traces of ω- and γ-gliadin peptides did not elicit a significant interferon (IFN)-γ response in T-cell assays, suggesting effective detoxification of gluten-derived immunogenic sequences. PEPs from *Sphingomonas capsulata* have also been optimized using machine learning to improve stability and activity under gastric conditions, with promising mutant variants demonstrating enhanced gluten hydrolysis and potential for oral enzymatic therapy [75].

## Repurposing microbial and plant proteases for therapeutic applications

Microbial and plant proteases are attractive therapeutic agents due to their cost-effectiveness, stability, and ease of production compared with animal-derived enzymes [76,77]. Recent advances highlight their potential across diverse medical applications, including neurodegenerative diseases, metabolic disorders, and viral infections. Harnessing their enzymatic capabilities may enable novel treatments for conditions currently lacking effective therapies. Several of these enzymes have already advanced into clinical use or preclinical evaluation (Table 3). The following examples highlight microbial and plant proteases, outlining their proposed mechanisms and therapeutic benefits.

**Table 3:** Selected microbial and plant proteases with therapeutic applications

Protease source	Therapeutic application	Key outcome/benefit	Reference
*Zingiber officinale* (Zingipain)	Alzheimer’s disease	Acetylcholinesterase inhibition	[78]
	Type 2 diabetes	Dipeptidyl peptidase-IV inhibition	[80]
*Penicillium janthinellum*	Antiviral (SARS-CoV-2)	Potential viral protein degradation	[83]
*Clostridium histolyticum* (collagenase)	Wound debridement	Accelerated healing; selective collagen degradation	[85]
*Bacillus subtilis* (Nattokinase)	Cardiovascular, anti-inflammatory	Fibrinolytic and anti-inflammatory activity	[86,89]

Proteases from *Zingiber officinale* (ginger) show promise as acetylcholinesterase inhibitors, potentially providing symptomatic relief in Alzheimer’s disease (AD) with superior stability across temperature and pH ranges compared with current drugs [78,79]. Additionally, ginger proteases hydrolyze wheat gluten to produce bioactive peptides that inhibit dipeptidyl peptidase-IV (DPP-IV), a target in type 2 diabetes management [80]. These peptides demonstrate potent inhibitory activity and improve gluten solubility and bioavailability. While some peptide modifications reduce DPP-IV inhibition, they may confer other health benefits, such as hepatoprotection and mood regulation [81].

Microbial proteases, especially extracellular fungal enzymes, demonstrate potential antiviral activity by targeting key viral proteins [82]. *In silico* docking studies have demonstrated that proteases from the A1A, M20A, S10, S8A, and T1A families can bind to key SARS-CoV-2 viral proteins, such as the spike, envelope, ORF-7a, and Nsp2, with binding energies below −50 kJ/mol [83]. Additionally, clinically approved microbial proteases, like *Clostridium histolyticum* collagenase, are already used in enzymatic wound debridement and treating fibrotic diseases [84,85].

Lastly, nattokinase from *Bacillus subtilis* exhibits fibrinolytic and anti-inflammatory activities [86,87]. Directed evolution and DNA family shuffling produced mutants with up to 2.3-fold increased catalytic efficiency [88]. In a separate study, the acidic stability of nattokinase was enhanced through pH-induced buffering and mutagenesis, identifying mutations that improved acid resistance and extended half-life by three-fold, though with slightly reduced activity [89].

## Protease-mediated degradation of amyloidogenic proteins

**Figure 3:**
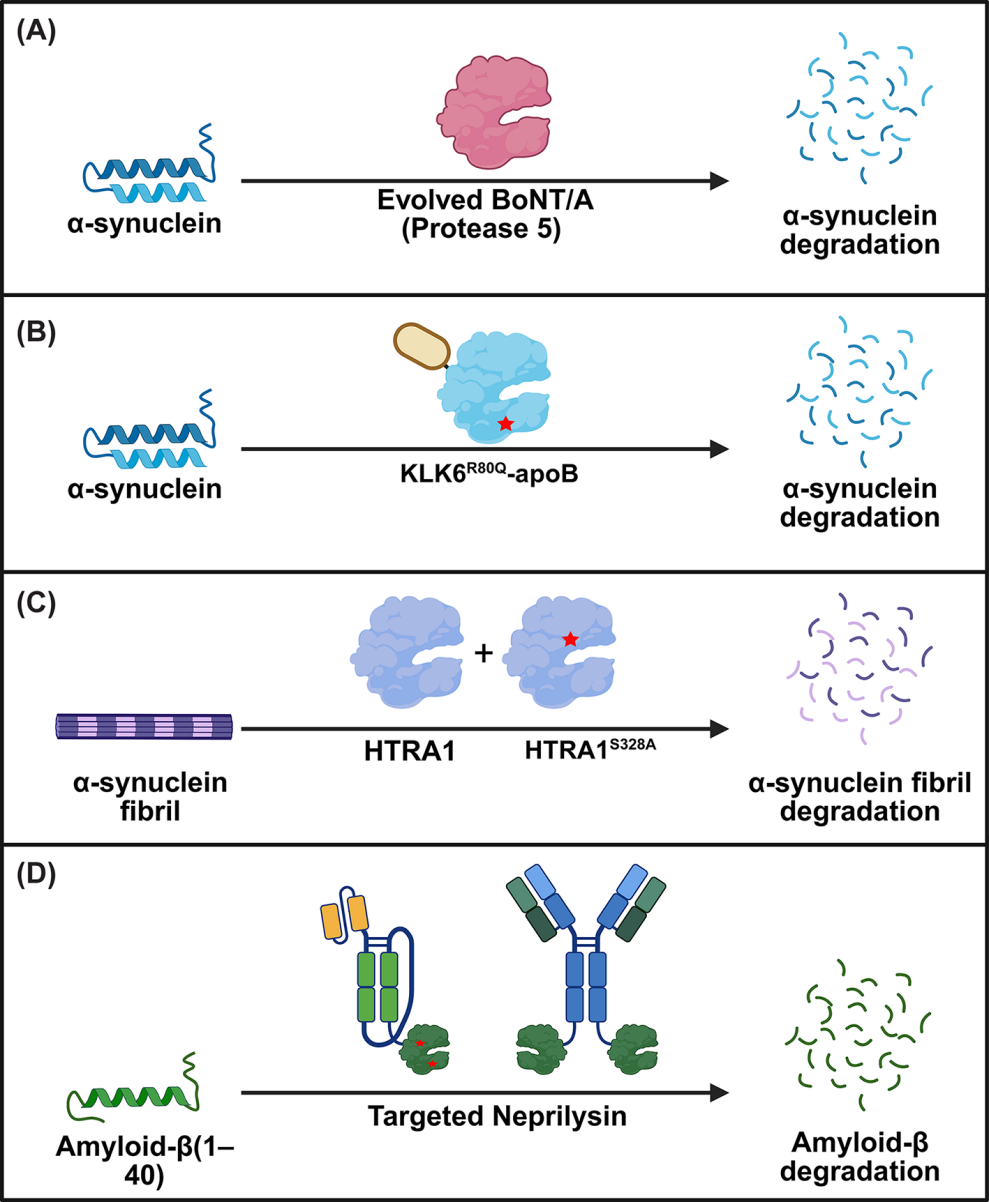
Strategies for the proteolytic degradation of amyloidogenic proteins. (**A**) A BoNT/A variant (Protease 5), generated through structure-guided mutagenesis, exhibits high catalytic activity and selectivity for α-synuclein (α-syn). (**B**) The highly stable KLK6R80Q variant is fused to a brain-targeting tag (apoB) and effectively reduces α-syn accumulation. (**C**) Pre-treatment of α-syn fibrils with the inactive HTRA1 variant, HTRA1S328A, exposes previously inaccessible epitopes, allowing active HTRA1 to cleave and degrade pre-formed fibrils. (**D**) Several targeted-Neprilysin formats, including scFv-NEP and antibody-NEP, guide catalytic activity to degrade amyloid-β. Abbreviations: KLK6, kallikrein-6; ApoB, apolipoprotein B; HTRA1, high-temperature requirement serine protease A1; BBB, blood–brain barrier.

Another area where proteases could unearth new opportunities and fulfill critical needs is in the degradation of aggregated proteins in amyloidosis. Amyloidosis is associated with a broad spectrum of diseases, including AD and Parkinson’s disease (PD), in which amyloid fibrils—insoluble, β-sheet-rich protein aggregates—accumulate, causing cardiomyopathy, neuropathy, and progressive neurodegeneration [90–92]. These fibrils resist natural clearance, and current treatments primarily slow progression and manage symptoms rather than removing the aggregates [93–95]. Antibody-mediated clearance of protein aggregates, while promising in preclinical settings, has had limited success in patients [96]. The dense packing of β-sheets within Aβ fibrils limits the accessibility of canonical protease cleavage sites, impeding proteolysis; however, cleavage near the termini has been shown to significantly destabilize the fibril structure [8,97–100]. This recognition has spurred interest in targeted proteolysis as a strategy to dismantle pathogenic aggregates (Figure 3).

### Proteolytic degradation of misfolded α-synuclein as a possible treatment avenue against synucleopathies

Synucleinopathies are a class of neurodegenerative disorders characterized by the accumulation of α-syn in neurons and glia [101]. When dysregulated, α-syn aggregates spread in a prion-like manner, disrupting neuronal synaptic vesicle trafficking and promoting soluble N-ethylmaleimide-sensitive factor attachment protein receptor complex assembly [102,103]. Targeted degradation of α-syn is therefore an attractive therapeutic goal.

One promising avenue is the reprogramming of proteases to selectively cleave α-syn. A notable example is the catalytic light chain of botulinum neurotoxin A (BoNT/A), a zinc metalloprotease with exceptional sequence specificity for its natural substrate SNAP25. Despite minimal homology between SNAP25 and α-syn, BoNT/A’s extended (>30 amino acid) substrate-binding interface and structurally distinct catalytic and substrate-recognition sites were leveraged to identify an α-syn target site in the non-amyloid core (NAC) region between Q79 and K80 [104]. Through a stepwise, structure-guided mutagenesis campaign spanning 45 sites, a novel BoNT/A variant (Protease 5) was generated with high catalytic activity and selectivity for α-syn. Protease 5 efficiently depleted overexpressed α-syn in mammalian cells while maintaining minimal off-target activity. Importantly, this work establishes a broadly adaptable framework for reprogramming BoNT/A to target other intrinsically disordered proteins implicated in disease.

Another approach for clearing extracellular α-syn aggregates involves the serine protease kallikrein-6 (KLK6, neurosin), which endogenously degrades α-syn. However, KLK6 has many physiological substrates, including protease-activated receptors and glutamate receptors [105]. This unoptimized substrate specificity raises off-target concerns [106,107]. Engineering KLK6 for improved stability and α-syn selectivity has shown therapeutic potential. For example, an R80Q variant with extended half-life and an apolipoprotein B (apoB) brain-targeting tag reduced α-syn accumulation in oligodendrocytes and astrocytes, improved myelination, and ameliorated behavioral deficits in a multiple system atrophy mouse model [108]. Whether KLK6 can be evolved for dramatically improved α-syn potency and selectivity remains to be determined.

HTRA1, a trimeric enzyme with N-terminal, protease, and PDZ domains, can proteolyze and inhibit the aggregation of multiple aggregation-prone proteins, including α-syn, tau, TDP-43, and FUS [97,109]. This chaperone-like “disaggregase” activity is independent of ATP and, surprisingly, can be enhanced by catalytic inactivation (HTRA1^S328^), which more effectively suppresses α-syn seeding in cells and neurons [97]. HTRA1 engages the α-syn NAC domain within fibrils, solubilizing it and exposing protease-accessible sites. Although it also acts on other amyloidogenic proteins, its potency is greatest for α-syn, and it does not target well-folded proteins. Given its broad substrate range, therapeutic strategies may favor protease-inactive or engineered variants to selectively promote disaggregation while avoiding the degradation of native proteins, making HTRA1 a promising starting point for engineering therapeutics to treat amyloid-related diseases.

### Antibody-guided neprilysin for targeted amyloid-β degradation

Accumulation of amyloid-β (Aβ) species and plaques is considered to be a driver of AD pathogenesis [110]. An effective protease-based therapeutic against Aβ would need to cross the blood–brain barrier (BBB), achieve sufficient brain retention, and cleave only the target protein. Two different efforts using Neprilysin (NEP) underscore these strategies. NEP, a membrane-bound metallopeptidase, degrades Aβ but has broad substrate specificity that raises concerns about off-target effects [111,112]. Engineering efforts yielded muNEP (G399V/G714K), which showed 20-fold higher activity on Aβ(1–40) and a 3200-fold reduction in off-target cleavage (assessed across a panel of 16 known physiological peptides, including insulin, glucagon, and neurokinins) relative to wild-type NEP [113]. Despite these improvements, intravenous administration of NEP showed no effect on brain Aβ levels, underscoring the need for better transport through the BBB and improved retention at the target site [113].

To enhance NEP's ability to cross the BBB, researchers fused a transferrin receptor-binding moiety (scFv8D3) to a soluble form of NEP (sNEP) and muNEP, along with an Fc-based antibody fragment (scFc) to extend blood half-life [114]. These fusions achieved a prolonged blood half-life and approximately 20-fold greater brain uptake compared with soluble NEP alone. While the engineered proteins reduced monomeric and oligomeric Aβ levels in tg-ArcSwe mice, brain concentrations declined rapidly, indicating limited retention. Notably, the muNEP fusion failed to degrade wild-type-Aβ at higher Aβ concentrations (2.5 μM). This is likely this is because Aβ concentration was high enough for aggregation to occur, and muNEP has a strong preference for cleaving Aβ at position 20-21, a site that becomes inaccessible in Aβ aggregates. Together, these findings demonstrate that while antibody-guided BBB transport can substantially improve brain delivery of proteases, there is still a need for improved retention.

In an effort to increase the specificity of NEP for Aβ Romei and colleagues fused NEP to crenezumab, an Aβ-specific antibody, producing a C-terminal fusion with 15-fold higher potency for Aβ (1–40) and 9-fold higher potency for Aβ(1–42) compared with untargeted NEP [8]. Interestingly, faster antibody off-rates correlated with greater enzymatic cleavage, suggesting that optimizing binding kinetics could further boost efficacy. While promising, *in vivo* BBB penetration for such fusions remains untested. More broadly, this study illustrates how antibody–protease fusions can address key limitations of proteases as therapeutics by conferring increased target specificity while preserving the catalytic, sub-stoichiometric turnover that distinguishes enzymes from binding-based modalities.

More broadly, fusion of proteases to targeting domains such as antibodies or nanobodies represents a powerful strategy to enhance substrate specificity and localize catalytic activity. Nb–protease fusions, in particular, offer several advantages, including small size, high stability, and ease of genetic fusion [115]. However, their development introduces several engineering challenges. Fusion can impose steric constraints that reduce catalytic efficiency or limit substrate access, necessitating careful optimization of linker length, flexibility, and fusion orientation [116]. In addition, maintaining the structural stability of both the protease and targeting domain is critical, as misfolding or aggregation can compromise activity and manufacturability. From a production standpoint, while nanobodies are generally amenable to high-yield expression in microbial systems, the inclusion of protease domains and post-translational requirements may necessitate more complex expression platforms, increasing the cost of goods relative to simpler biologics. Despite these challenges, advances in protein engineering and expression technologies are steadily improving the scalability and clinical feasibility of these multifunctional constructs.

## Protease-mediated inhibition of upper respiratory viral propagation

ColdZyme is a medical device mouth spray developed by Enzymatica AB, designed to reduce viral propagation in the upper respiratory tract by protecting airway epithelial integrity and limiting viral release from infected cells [117]. Most upper respiratory tract infections (URTIs) are initiated in the mucus of the nasopharynx before spreading throughout the nasal cavity, suggesting that early inhibition of viral propagation in the pharyngeal region during the incubation phase may shorten UTRI duration [118]. ColdZyme consists of a hyperosmotic glycerol solution containing a cold-adapted trypsin derived from Atlantic cod (*Gadus morhua*), which forms a temporary barrier when sprayed onto the back of the mouth. The proposed mechanism is that viral particles become physically trapped within this barrier and are subsequently inactivated, in part through trypsin's cleavage of viral surface proteins required for receptor binding and host cell entry [119]. This mechanism has not yet been proven experimentally.

*In vitro* virucidal assays demonstrate that ColdZyme inactivates multiple respiratory viruses, including rhinovirus types 1A and 42, influenza A virus (H3N2), respiratory syncytial virus, adenovirus type 2, and coronaviruses [119,120]. Early clinical evaluation included a 5-month open-label study in elderly care facility personnel, which reported a reduction in sick-leave days from 5.2 to 3.7 days (29%) during the ColdZyme use period compared with a control period (*P* = 0.054), with 63% of participants reporting milder symptoms than during previous colds [121]. More recent studies have examined ColdZyme efficacy under free-living conditions in endurance athletes, a population at elevated risk of URTI. In a 3-month randomized trial comparing ColdZyme to no treatment, illness incidence did not differ between groups; however, URTI episode duration was significantly shorter with ColdZyme (7.7 ± 4.0 versus 10.4 ± 8.5 days; *P* = 0.016) [118]. A subsequent randomized, double-blind, placebo-controlled trial in active athletes further demonstrated reductions in symptom duration (mean reduction ∼5 days), missed training days (mean reduction ∼2.4 days), and symptom severity [117]. Importantly, pathogen-specific analyses indicated that rhinovirus load was markedly reduced by ColdZyme treatment, whereas *Haemophilus influenzae* was less responsive, suggesting differential efficacy depending on the causative agent. Consistent with these clinical findings, *in vitro* studies using differentiated primary human airway epithelial cultures showed that a single prophylactic application of ColdZyme significantly reduced apical and basolateral release of two non-enveloped rhinovirus strains (RV35 and RV48), preserved transepithelial electrical resistance, and protected epithelial architecture from virus-induced damage. Importantly, there was no difference between groups on URTI incidence, even when subjects used the product preventatively.

Collectively, these data indicate that ColdZyme does not prevent URTI acquisition but instead limits local viral propagation and epithelial damage, resulting in reduced symptom severity and illness duration, particularly in rhinovirus-driven infections. Future work could examine whether efficacy can be further improved by replacing the cod-derived trypsin with an engineered protease optimized to cleave conserved viral surface proteins involved in host cell entry.

## Conclusion and future directions

Across immune modulation, infectious disease, metabolic disorders, and neurodegeneration, proteases are rapidly emerging as a versatile therapeutic class. The sections of the present review highlight a consistent theme: despite diverse origins and mechanisms, therapeutic proteases face common barriers such as immunogenicity, limited half-life, insufficient specificity, and challenges in targeted delivery that have historically constrained their clinical use. These limitations have led to proteases being viewed as niche or high-risk agents relative to antibodies and small molecules. However, this perception is becoming increasingly outdated.

Immunomodulatory proteases, such as IgG- and IgM-cleaving enzymes, show strong clinical potential but require innovations that mitigate immunogenicity, preserve protective antibodies, and enable repeat dosing. To facilitate broader clinical application, engineering strategies including humanization, de-immunization, glycosylation, and zymogen design are being developed to improve enzyme stability, reduce immunogenicity, and enable repeated dosing. Successfully addressing these challenges will be crucial to unlocking the full potential of protease-based immune modulation across autoimmune diseases, transplantation, gene therapy, and beyond.

Microbial and plant proteases exhibit broad antiviral activity, metabolic modulation, and toxin degradation. Yet translating these enzymes into medicines demands improved substrate specificity, enhanced stability, and formulations that preserve activity *in vivo*. Advances in metagenomics, directed evolution, and stability engineering promise to unlock far more of this molecular diversity for therapeutic use.

Proteases targeting misfolded or aggregated proteins illustrate the unique power of enzymes to remodel pathogenic structures rather than merely manage symptoms. Key challenges remain—particularly precision, delivery across the blood-brain barrier, and avoidance of off-target degradation—but the field is advancing toward multifunctional, highly selective proteases guided by nanobodies, engineered domains, or conditional activation mechanisms.

The ability to reprogram proteases for precise therapeutic applications represents a transformative approach in protein engineering. By integrating directed evolution, substrate specificity tuning, and innovative targeting strategies, researchers have made significant progress in enhancing the clinical utility of proteases. The engineering of BoNT and nattokinase demonstrates how modifications can enhance catalytic properties and stability, while the development of proteases targeting neurodegenerative aggregates underscores the potential to treat protein misfolding disorders.

Despite these advances, significant hurdles remain. One of the key challenges is achieving targeted activation, i.e., developing proteases that remain inactive until they reach their intended substrate or location, thereby minimizing off-target effects. Inspired by systems like CRISPR–Cas, in which enzymatic activity is triggered by specific binding events, future efforts may focus on designing allosterically controlled proteases that activate only upon binding their pathological target. This approach could dramatically enhance therapeutic specificity while preserving normal physiological functions.

Beyond intrinsic control strategies, Nb-guided protease modulation represents a rapidly emerging approach for extrinsic regulation of protease activity and substrate selectivity. By binding to noncatalytic or regulatory regions, Nbs can fine-tune protease function rather than abolish it, offering a level of precision difficult to achieve with traditional inhibitors. For example, activated protein C, llama-derived Nbs were identified that selectively recognize the active protease over its zymogen and bias its multifunctional activity toward cytoprotective signaling while preserving coagulation balance [122]. Similarly, furin-specific Nbs isolated via phage panning bind distal domains such as the P-domain, sterically restricting access of large pathogenic substrates like diphtheria toxin without broadly suppressing proteolytic activity [123,124].

A particularly important limitation for protease therapeutics is restricted access to intracellular targets and the central nervous system. Most proteases are large, hydrophilic macromolecules that lack intrinsic membrane permeability, confining their activity primarily to extracellular substrates [125]. Similarly, the BBB presents a formidable obstacle, preventing passive diffusion of proteases into the brain. Consequently, the successful deployment of therapeutic proteases requires engineering delivery strategies alongside catalytic function. Strategies to overcome these barriers include receptor-mediated transcytosis, nanoparticle-based delivery systems, and fusion to BBB-shuttling domains [126]. Notably, an inactivated botulinum neurotoxin (BoNT) platform has been repurposed for Nb-guided, cell-type-specific cytosolic delivery of protein cargos by replacing its native receptor-binding domain with nanobodies directed against selected surface markers [127]. By Retaining BoNT's efficient endosomal escape machinery while eliminating toxicity, this system demonstrates how toxin-derived scaffolds can be repurposed for precise and programmable intracellular delivery of therapeutic proteases.

As synthetic biology, computational protein design, and high-throughput screening continue to converge, the future of protease therapeutics lies in creating enzymes that are not only potent and specific but also programmable and regulatable. Such innovations promise to redefine therapeutic paradigms across neurodegeneration, thrombosis, autoimmune disease, and beyond.

## Data Availability

Data will be made available upon request.

## Open Access

Open access for this article was enabled by the participation of the University of Florida in an all-inclusive Read & Publish agreement with Portland Press and the Biochemical Society.

## Abbreviations

α-syn: α-synuclein
AD: Alzheimer’s disease
ADA: anti-drug antibodies
agIgA1: aberrantly glycosylated IgA1
anti-GBM: anti-glomerular basement membrane
apoB: apolipoprotein B
Aβ: amyloid-β
BBB: blood-brain barrier
BoNT: botulinum neurotoxin
BoNT/A: botulinum neurotoxin A
CeD: Celiac disease
CLN2: ceroid lipofuscinosis type 2
DPP-IV: dipeptidyl peptidase-IV
DSAs: donor-specific antibodies
E40: endoprotease 40
EMA: European Medicines Agency
FcRn: neonatal Fc receptor
FDA: Food and Drug Administration
FIX: f actor IX
FVIII: factor VIII
HLAs: human leukocyte antigens
HTRA1: HtrA serine peptidase 1
IceM: IgM-specific protease from *Lachnoanaerobaculum saburreum*
IdeS: (imlifidase) IgG-degrading cysteine endopeptidase from *Streptococcus pyogenes*
IFN: interferon
IgA: immunoglobulin A
IgE: immunoglobulin E
IgG: immunoglobulin G
IgM: immunoglobulin M
IND: Investigational New Drug
KLK6: kallikrein-6 (or neurosin)
MarzAA: Marzeptacog alfa
NAC: non-amyloid core
Nb: nanobody
NEP: Neprilysin
PD: Parkinson’s disease
PEPs: prolyl endopeptidases
RA: rheumatoid arthritis
rAAV: recombinant adeno-associated virus
ScFc: Fc-based antibody fragment
SLE: systemic lupus erythematosus
sNEP: soluble form of neprilysin
TPP1: tripeptidyl-peptidase 1
URTIs: upper respiratory tract infections
VWF: von Willebrand factor
